# Scleral Buckling with Viscoelastics or Gas Injection for Bulging Retinal Detachments: A Retrospective Cohort Study

**DOI:** 10.1155/2021/6694199

**Published:** 2021-04-08

**Authors:** Quan-Yong Yi, Wen-Die Li, Qian Gui, Sang-Sang Wang, Li-Shuang Chen, Jing-Hai Mao, Yan Gong, Xia-Jun Wang

**Affiliations:** Ningbo Eye Hospital, Ningbo, China

## Abstract

**Objective:**

To examine the use of a viscoelastic agent instead of air in the vitreous cavity during surgery for scleral buckling.

**Methods:**

This was a retrospective cohort study of patients who underwent scleral buckling surgery for bulging rhegmatogenous retinal detachment (RRD) at Ningbo Eye Hospital from 07/2016 to 12/2019. The patients were grouped into drainage, air injection, cryotherapy and explant (DACE) and drainage, viscoelastic injection, cryotherapy, and explant (DVCE) groups, which were comparatively assessed.

**Results:**

There were 25 and 22 patients in the DVCE and DACE groups, respectively. The surgery was significantly shorter with DVCE than DACE (*P* < 0.05), with less intraoperative external pressure adjustment (*P* < 0.05). BCVA was lower in the DVCE group at 1 week compared with the DACE group (*P* < 0.05). Successful retinal reattachment was observed in 92.0% and 81.8% of the DVCE and DACE groups, respectively (*P* < 0.05). Cases requiring laser replenishing after the operation were less in the DVCE group compared with the DACE group (*P* < 0.05). There were no differences in complications and intraocular pressure between the two groups (all *P* < 0.05).

**Conclusion:**

DVCE has better operative characteristics and faster vision recovery than DACE, with similar outcomes.

## 1. Introduction

Retinal detachment is the separation of the neurosensory retina from the underlying retinal pigment epithelium [1, 2]. Primary retinal detachment refers to rhegmatogenous retinal detachment (RRD) that occurs due to holes, tears, or breaks in the retina of a previously uninvolved eye with the lens present without complicating factors (e.g., underlying diabetic retinopathy and penetrating trauma) or cataract surgery performed, usually with artificial intraocular lens [1, 3]. RRD affects one in 10,000 individuals annually [1, 2], with an estimated lifetime risk of 3% by 85 years of age [1].

The key treatment of RRD is to close the hole. Many RRD cases can be managed successfully without subretinal fluid (SRF) drainage, and scleral buckling surgery is the classic treatment approach for RRD [4, 5]. For bulging retinal detachment with a high degree of retinal bulge, subretinal fluid release is still required to promote retinal reattachment and accurately locate the hole perioperatively. Nevertheless, in traditional buckling surgery, after releasing the subretinal fluid, intraocular pressure decreases, and it is necessary to inject gas into the vitreous cavity for increasing the intraocular pressure to complete the operation. For scleral buckling surgery of highly bulging retinal detachment, there are two classic surgical methods. The first is cryotherapy, drain, air injection, and explant (CDAE). This method has two shortcomings: (1) as the bulging retina sometimes has a high degree of swelling, the gap between the choroid and the retina is wide when the fluid is not discharged, which makes it impossible to perform smooth condensation and positioning; (2) liquid discharge after condensation increases the risk of subchoroidal bleeding. The second method for bulging retinal detachment is drainage, air injection, cryotherapy, and explant (DACE). This method consists of draining the subretinal fluid first, which leads to retinal reattachment, and then injecting air to maintain intraocular pressure to complete the operation [6, 7]. However, it also has some shortcomings: after injecting air, the density and refractive index of the air are quite different from those of the vitreous in the eye, which affects the observation of subsequent operation steps such as retinal tear positioning and condensation [8, 9]. Based on the above shortcomings, novel and effective approaches are needed for scleral buckling surgery of highly bulging retinal detachment.

Viscoelastic agents are commonly used as auxiliary materials in ophthalmic surgery, especially cataract and vitreous surgery [10–12]. Comparing phacoemulsification surgery (PE) with viscosurgical devices (OVDs) and without OVD, best-corrected visual acuity (BCVA) values were significantly different on the day following surgery (0.41 ± 0.26 and 0.54 ± 0.34 logMAR) in OVD and non-OVD groups, respectively, although both groups had similar values at 6 months postoperatively [12]. The density and refractive index of a viscoelastic agent are basically close to those of the vitreous [13–15]. Recently, local dry vitrectomy combined with segmental scleral buckling and viscoelastic tamponade was evaluated for treating partial rhegmatogenous retinal detachment (RRD) with local vitreous traction, and postoperative visual acuities showed improvement or remained stable in the totality of patients, with no major complications besides transient mild intraocular pressure increased in 3 of 11 patients [16]. Accordingly, we hypothesized that, unlike air, a given viscoelastic agent should have no or little effects on the location of the hole and the condensation treatment.

**Figure 1:**
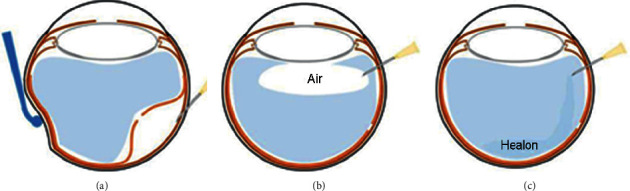
Schematic diagram of extrascleral drainage (a), air injection into the vitreous cavity (b), and viscoelastic agent injection into the vitreous cavity (c).

Therefore, this study aimed to examine whether the use of a viscoelastic agent instead of air in the vitreous cavity during surgery for scleral buckling could achieve good outcomes. We found that drainage, viscoelastic injection, cryotherapy, and explant (DVCE) (Figure 1) is superior to DACE in terms of operative characteristics, allowing faster vision recovery with similar outcomes in the management of RRD.

## 2. Materials and Methods

### 2.1. Study Design

This was a retrospective cohort study of patients who underwent scleral buckling surgery for bulging RRD at Ningbo Eye Hospital from July 2016 to December 2019. This study complied with the Declaration of Helsinki and was reviewed and approved by the Ethics Committee of Ningbo Eye Hospital (no. 20200311).

Inclusion criteria were as follows: (1) bulging RRD; (2) RRD with equatorial and anterior hiatus requiring scleral buckling surgery; (3) bulging RRD requiring SRF drainage; (4) vitreous in good condition (no bleeding or severe turbidity); (5) proliferative vitreoretinopathy (PVR) grades A to C; and (6) hole size of 1 to 4 DD, but with the possibility to close the lower serrated edge to 1 quadrant. Exclusion criteria were as follows: (1) planned vitrectomy or a history of vitreoretinal surgery; (2) fundus not seen due to severe turbidity of the refractive interstitium; (3) a history of uveitis; and (4) a history of trauma.

### 2.2. Preoperative and Postoperative Examinations

All patients underwent naked eye vision, corrected vision, intraocular pressure, refractive status, slit lamp microscope, fundus, Aalborg photography, eye B-mode ultrasound, and optical coherence tomography (OCT) examinations. Break location and number and the extent of peripheral retina and macular detachment were determined by front lens, trimirror, and indirect ophthalmoscopy. During the operation, surgery time (from the cutting of the conjunctiva to the final suture), the position of the scleral puncture, use of cerclage as well as silica gel and silicone sponge, and anesthesia were recorded.

BCVA was measured by the international standard visual acuity chart. For statistical analysis, BCVA was converted into logarithm of the minimum angle of resolution (LogMAR) values. LogMAR = l g (1/decimal visual acuity). The index was converted to 1.86; the manual conversion was 2.28, and light perception was converted to 2.84.

### 2.3. Surgical Process

The ophthalmologists discussed with the patient about the pros and cons of DACE and DVCE, and the surgical approach was finally selected by the patient. The two surgical procedures were similar, except that DACE used air for injection, while DVCE used viscoelastic injection (Figure 1).

Before the operation, all patients underwent full mydriasis with Medori eye drops and 1% atropine eye drops, followed by 2% lidocaine and 0.75% bupivacaine for anesthesia. All operations were performed by the same surgeon (Dr. Y.Q.Y). All patients underwent scleral buckling surgery. Before surgery, systemic and eye infections were ruled out and controlled, and blood glucose and blood pressure were controlled. A HEINI indirect ophthalmoscope, a Suzhou Xinmingren carbon dioxide condenser, a silica gel strip, a silica gel block, and a silicon sponge were used. After routine disinfection and anesthesia, according to hiatus' position and the scope of external compression, the bulbar conjunctiva was cut along the corneoscleral limbus. The corresponding extraocular rectus muscles were separated and pulled.

**Figure 2:**
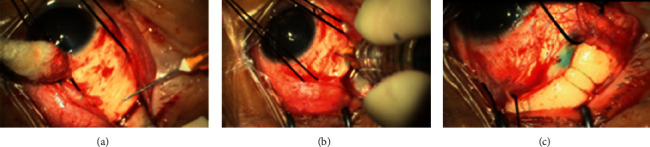
DVCE procedure. (a) Drainage. Puncture and drainage of the scleral wall corresponding to the highest point of the retinal bulge. (b) Injection of the viscoelastic agent. After supplying the subretinal fluid, a puncture was made 4 mm behind the limbus of the cornea and into the vitreous cavity to inject the viscoelastic agent for increasing intraocular pressure. (c) External pressure (explant). A suitable silicon sponge block or silicone tape was placed at the position of the hole.

For DVCE, a 27-G needle was used to puncture the scleral wall at a 30-degree angle at the highest point of the retinal bulge. The depth was based on the feeling of the puncture and outflow of SRF. A retractor was used for pressing the eyeball wall to apply an appropriate pressure to maintain intraocular pressure and facilitate the outflow of SRF from the puncture port (Figure 2(a)). After draining the SRF, the vitreous cavity was punctured 4 mm behind the limbus of the cornea to inject the viscoelastic agent (VisCoat, Alcon, Hunenberg, Switzerland) to the intraocular pressure T_−1_ (Figure 2(b)). For DACE, all steps were the same as described for DVCE, except that air was injected instead of the viscoelastic agent. After draining the SRF, the vitreous cavity was punctured 4 mm behind the limbus of the cornea, and sterile air was injected into the intraocular pressure T_n_.

After injection of the viscoelastic agent or sterile air, a CO_2_ condensing pen was used to press and condense the edge of the hole with live observation under an indirect ophthalmoscope. When the retina at the edge of the hole became white, the condensation process was stopped, and trypan blue was applied. A suitable silicon sponge block or circling silicone tape as an explant was sewn at the position marked by the puncture (Figure 2(c)).

At the end of all the surgical procedures, dexamethasone (5 mg/1 mL) was injected into the inferior subconjunctival space. During follow-up, antiglaucoma eye drops such as *β*-blockers, carbonic anhydrase inhibitors, and prostaglandin analogs, were prescribed with IOP above 21 mmHg.

### 2.4. Outcome and Follow-Up

On the day after the operation, retinal reattachment, vision, and intraocular pressure were assessed. Complications were recorded: hole not closed by 1 week to 1 month after the operation, remaining SRF, unresolved retina detachment, and subretinal hemorrhage (subretinal distribution and size graded as less or greater than 1 disk area (<1 DA or >1 DA)). BCVA, slit lamp microscopy findings, intraocular pressure, fundus examination using the Optomap panoramic eye scan system, and OCT data were reviewed at 1 week, 1 month, 3 months, and 6 months after surgery.

### 2.5. Statistical Analysis

Statistical analysis was performed with SPSS 17.0 (SPSS Inc., Chicago, USA). Normally and nonnormally distributed continuous variables were presented as mean ± standard deviation (SD) and median (range), respectively, and compared by Student's *t*-test and the Mann–Whitney *U*-test, respectively. Categorical data were presented as *n* (%) and assessed by the chi-square test or Fisher's exact test, as appropriate. Two-sided *P* < 0.05 was considered statistically significant.

## 3. Results

### 3.1. Characteristics of the Patients

There were 25 patients in the DVCE group (25 eyes), including 17 males and eight females. There were 22 patients (22 eyes) in the DACE group, including 15 males and seven females. The patient characteristics are presented in Table 1. There were no significant differences between the two groups in age, sex, disease course, BCVA, and affected quadrants.

### 3.2. Intraoperative Characteristics

The surgery was significantly shorter with DVCE than DACE (38 ± 16 vs. 49 ± 15 min, *P* < 0.05) (Table 2). Intraoperative excessive pressure adjustment was observed in one patient of the DACE group and four patients of the DVCE group (*P* < 0.05). There were no significant differences in combined cerclage and subretinal hemorrhage (both *P* < 0.05). There were no iatrogenic retinal injuries.

### 3.3. BCVA after Surgery

Table 3 presents BCVA values after surgery. BCVA was lower in the DVCE group at 1 week compared with the DACE group (1.5 ± 0.5 vs. 2.0 ± 0.5, *P*=0.002). There were no significant differences in BCVA between the DVCE and DACE groups at 1, 3, and 6 months (all *P* < 0.05).

### 3.4. Follow-Up

There were no differences in IOP between the two groups at 1 week and 1, 3, and 6 months (all *P* < 0.05) (Table 4). Successful retinal reattachment was observed in 92% and 82% patients of the DVCE and DACE groups, respectively (*P* < 0.05). Cases requiring laser replenishing after the operation were less in the DVCE group compared with the DACE group (*P* < 0.05). There were no differences in reoperation rate between the two groups.

## 4. Discussion

The use of air in the classical DACE surgery for RRD has disadvantages associated with the difference in refractive index between air and vitreous, and the gas injected into the vitreous cavity, which is usually located behind the lens, also affects observation [8, 9]. Therefore, this study aimed to examine the use of a viscoelastic agent instead of air in the vitreous cavity during surgery for scleral buckling. The results suggested that DVCE has better operative characteristics and faster vision recovery than DACE, but similar outcomes, suggesting that viscoelastic agent use may be a new option for the treatment of bulging RRD.

The retinal hole is the main cause of retinal detachment, and the purpose of curing retinal detachment can be achieved by sealing the hole [17]. Although the related surgical methods have been continuously improved, the basic principle remains to close the hiatus, with minimal trauma, low economic cost, and relatively simple operation. Scleral buckle surgery has been applied in clinical practice for nearly a hundred years because of minimal trauma, low cost, good curative effect, and few postoperative complications. For bulging retinal detachment, it is necessary to remove the SRF to facilitate retinal reattachment and condensation. On the other hand, removing the SRF decreases intraocular pressure, and injection of auxiliary substances into the vitreous cavity is therefore required to maintain intraocular pressure and to apply pressure on the retina to facilitate reattachment [6, 7]. Previously, the use of perfluoroalkanes has been described [18]. The traditional scleral buckling surgery generally uses sterile air into the vitreous. However, the density and refractive index of air are quite different from those of the vitreous in the eye, which could affect the observation of subsequent operations such as retinal hole positioning and condensation; this might lead to complications or lower the success rate of the operation. Indeed, cataract, secondary glaucoma, and retinal artery occlusion have been reported with the use of gas [19, 20]. In addition, when the patient switches from the supine position to the upright position after the operation, the gas in the vitreous cavity moves upward, which pulls the lower vitreous and the retina, potentially generating new holes. Newly generated tears are one of the causes of failure after scleral buckling surgery. In the DACE group in this study, two of the four unsuccessful retina reattachment cases were due to newly created inferior tears.

Viscoelastic agents are commonly used as auxiliary materials in clinical ophthalmic surgery, including cataract and vitreous surgery [10–12]. The density and refractive index of a viscoelastic agent is close to those of the vitreous body [10–12], which allows a more precise observation of the subsequent hole positioning and condensation treatment after viscoelastic agent injection into the vitreous cavity. In addition, viscoelastic agents can liquefy and are gradually absorbed as the vitreous regenerates, and do not stay long in the eye [10, 21–23]. In this study, the operation time was significantly shorter in the DVCE group compared with the DACE group, probably because of better visualization and easier positioning. In addition, the patients in the DVCE group had better visual acuity at 1 week, but the difference was no more significant by 1 month. A previous study showed early visual recovery after using a viscoelastic agent, supporting the present findings [24]. In addition, a recent comparative multicenter study demonstrated that limited vitrectomy is time-efficient and effective in removing epiretinal membrane without additional complications compared to complete vitrectomy [25]. Another study reported good outcomes of sub-perfluoro-*n*-octane injection as a viscoelastic agent in patients with macular hole retinal detachment [26]. The rate of retinal reattachment was numerically higher, but the difference was not significant, probably because of the small number of patients. Animal studies showed high levels of anatomic reattachment after using a viscoelastic solution, supporting the present findings [14, 27, 28].

This study had limitations. It was a retrospective study, thus limited to the data available in medical charts. In addition, the sample size was small. Furthermore, different viscoelastic agents are available [24, 29, 30], and future studies could be designed to compare them. Moreover, a retrospective study recently showed that microscope-assisted *ab externo* surgery is effective and safe, reducing discomfort, allowing the surgeon to work with both hands free, and providing an optimal visualization of various surgical steps [25], which should be compared with the currently proposed approach in large multicenter prospective trials. Finally, follow-up was short, and the long-term effect of the viscoelastic agent should be examined.

## 5. Conclusions

In conclusion, DVCE has better operative characteristics and faster vision recovery than DACE, but similar outcomes, suggesting that viscoelastic agents for the treatment of bulging RRD have advantages over air. Additional studies are required to determine the optimal viscoelastic agent.

## Figures and Tables

**Table 1:** Characteristics of the patients.

Parameters	DVCE	DACE	*P*
Cases, *n*	25	22	
Age, mean ± SD, years	36.16 ± 16.23	33.39 ± 15.97	0.749
Sex (male/female)	17/8	15/7	0.813
Disease course, mean ± SD, months	3.12 ± 1.12	3.65 ± 1.49	0.376
Preoperative BCVA, logMAR ± SD	1.26 ± 0.78	1.39 ± 0.67	0.487
Intraocular pressure, median (range), mmHg	6–20	6–22	0.429
Average number of quadrants of retinal detachment range, *n* (%)			
1 quadrant	16 (64.0)	14 (63.6)	0.725
2-3 quadrants	7 (28.0)	7 (31.8)	0.481
All 4 quadrants	2 (8.0)	1 (4.5)	0.396
Macular detachment, *n* (%)	15 (60.0)	15 (68.2)	0.557
With lattice degeneration and cystic degeneration, *n* (%)	17 (68.0)	15 (68.2)	0.891

BCVA: best-corrected visual acuity; logMAR: logarithm of the minimal angle of resolution; DVCE: drainage, viscoelastic injection, cryotherapy, and explant; DACE: drainage, air injection, cryotherapy, and explant.

**Table 2:** Intraoperative characteristics.

Parameters	DVCE (*n* = 25)	DACE (*n* = 22)	*P*
Operation time, mean ± SD, min	38 ± 16	49 ± 15	<0.05
Combined cerclage, *n* (%)	2 (8.0%)	2 (9.1%)	0.965
Intraoperative external pressure adjustment, *n* (%)	1 (4.0%)	4 (18.2%)	<0.05
Subretinal hemorrhage, *n* (%)	1 (4.0%)	1 (4.5%)	0.891
Iatrogenic retinal hole, *n* (%)	0	0	0.875

DVCE: drainage, viscoelastic injection, cryotherapy, and explant; DACE: drainage, air injection, cryotherapy, and explant.

**Table 3:** Best-corrected visual acuity after the operation.

	1 week	1 month	3 months	6 months
DVCE group				
BCVA, logMAR ± SD	1.50 ± 0.45	0.89 ± 0.57	0.68 ± 0.61	0.59 ± 0.46

DACE group				
BCVA, logMAR ± SD	2.0 ± 0.52	0.95 ± 0.61	0.65 ± 0.51	0.62 ± 0.49
*P*	<0.05	0.219	0.746	0.482

BCVA: best-corrected visual acuity; logMAR: logarithm of the minimal angle of resolution; DVCE: drainage, viscoelastic injection, cryotherapy, and explant; DACE: drainage, air injection, cryotherapy, and explant.

**Table 4:** Postoperative characteristics.

Parameters	DVCE (*n* = 25)	DACE (*n* = 22)	*P*
Intraocular pressure			
1 week, median (range), mmHg	16.5 (6–38)	16.2 (7–36)	0.342
1 month, median (range), mmHg	15.9 (11–23)	15.7 (11–22)	0.261
3 months, median (range), mmHg	17.1 (9–23)	17.4 (11–24)	0.421
6 months, median (range), mmHg	15.9 (9–21)	16.1 (10–22)	0.518
Successful retinal reattachment, *n* (%)	23 (92.0%)	18 (81.8%)	<0.05
Retinal reattachment failure, *n* (%)	2 (8.0%)	4 (18.2%)	0.174
New holes	0	2 (9.1%)	
Missed holes	1 (4.0%)	1 (4.5%)	
Deviation of external pressure	1 (4.0%)	1 (4.5%)	
Scleral buckling again, *n* (%)	1 (4.0%)	3 (13.6%)	0.139
Vitrectomy again, *n* (%)	1 (4.0%)	1 (4.5%)	0.891
Need to replenish laser after the operation, *n* (%)	4 (16.0%)	4 (18.2%)	<0.05

DVCE: drainage, viscoelastic injection, cryotherapy, and explant; DACE: drainage, air injection, cryotherapy, and explant.

## Data Availability

No datasets were generated for this study.
